# Editorial: Business transformation through AI-enabled technologies

**DOI:** 10.3389/frai.2025.1577540

**Published:** 2025-03-11

**Authors:** Fethi Rabhi, Amin Beheshti, Asif Gill

**Affiliations:** ^1^School of Computer Science and Engineering, University of New South Wales, Kensington, NSW, Australia; ^2^School of Computing, Macquarie University, Sydney, NSW, Australia; ^3^School of Computer Science, University of Technology Sydney, Sydney, NSW, Australia

**Keywords:** AI in business, digital transformation, business process management (BPM), generative AI, business transformation, AI-enabled decision making

## 1 Introduction

The digital era is characterized by rapid technological advancements, with Artificial Intelligence (AI) emerging as a key driver of business transformation. Companies are increasingly integrating AI-enabled technologies into their operations to enhance productivity, streamline processes, and optimize decision-making (Beheshti et al., 2021). For instance, one of the Research Topic papers examined the integration of artificial intelligence in supply chain management (SCM) (Samuels). One of the key insights highlighted by this paper is that “integrating AI in SCM not only improves operational efficiency and sustainability but also promotes resilience against disruptions” (Samuels). From intelligent automation to predictive analytics, AI is reshaping industries by enabling organizations to leverage vast amounts of data for actionable insights. For instance, another Research Topic paper discussed the use of AI for the analysis of vast amount of data for generating and reporting software defects (Esposito et al.). This study reported several benefits such as rea-time analysis and operational efficiency which helped identifying and reducing the failure and errors in a timely manner. While AI-enabled automation offers several benefits, however, its inner working needs to be explained for enhancing stakeholders' trust. This topic was covered in this Research Topic by another accepted paper discussing the “stakeholder-centric explanations for black-box decisions: an XAI process model and its application to automotive goodwill assessments” (Haas et al.). Finally, the fourth paper in this Research Topic provided a methodology for the planning, implementation, and evaluation of skills intelligence management in the context of informed decision-making and adaptability (Kusmin et al.). Additionally, this editorial expands on these papers and draws our attention to one of the most significant advancements in AI which is the emergence of generative AI models, such as GPT and discusses its potential to revolutionize business process management (BPM) (Beheshti et al., 2023). By automating repetitive tasks, generating contextual insights, and facilitating seamless human-machine collaboration, AI-driven technologies are setting the foundation for intelligent business ecosystems. The remainder of this editorial further expands the topic of AI-enabled business process management followed by a discussion of key considerations. It concludes with a future outlook on the role of AI in continuous innovation.

## 2 AI in business process management

Business Process Management (BPM) is central to enterprise efficiency, governing how organizations design, analyze, and optimize workflows. Traditional BPM approaches relied on human expertise and structured methodologies. However, the integration of AI has ushered in a new era of smart BPM, where AI models automate process discovery, enhance workflow optimization, and provide intelligent recommendations.

For instance, ProcessGPT (Beheshti et al., 2023), an AI-driven BPM framework, leverages generative AI to streamline business processes. By analyzing historical data and learning from domain-specific knowledge, such technologies can generate process flows, identify inefficiencies, and recommend optimization strategies. The implications are profound: AI-powered BPM reduces operational costs, enhances agility, and enables organizations to adapt to evolving market conditions.

## 3 Data-centric and knowledge-intensive processes

AI's transformative impact extends to data-centric and knowledge-intensive processes, where decision-making is crucial. AI models can analyze vast datasets, detect patterns, and generate actionable insights, thereby augmenting human expertise. In domains such as finance, healthcare, and supply chain management, AI-driven analytics improve risk assessment, optimize resource allocation, and enhance customer experiences.

Moreover, knowledge-intensive industries, such as legal and research-driven enterprises, benefit from AI's ability to process complex information. By integrating AI models with knowledge graphs and semantic reasoning, businesses can enhance decision-making and foster innovation. AI-enabled knowledge management systems facilitate the retrieval of relevant information, automate document summarization, and support collaborative problem-solving.

## 4 AI-driven automation: from augmentation to full automation

One of the key drivers of business transformation is the shift from process augmentation to full automation. AI technologies are evolving from assisting human workers in decision-making to autonomously executing complex tasks. This transition is evident in various industries:

1. **Financial services**: AI-driven fraud detection systems analyze transactional data in real-time, identifying suspicious activities and preventing financial losses.
2. **Healthcare**: AI models assist medical professionals in diagnostics, drug discovery, and personalized treatment recommendations.
3. **Education**: AI-powered tools automate grading, generate personalized learning pathways, and enhance student engagement.
4. **Manufacturing**: AI-driven robotics and predictive maintenance optimize production lines, reducing downtime and improving efficiency.

As AI capabilities advance, businesses must strategically navigate the balance between human expertise and machine intelligence to maximize efficiency while ensuring ethical considerations and transparency in decision-making.

## 5 Challenges and ethical considerations

While AI presents unparalleled opportunities, it also raises challenges that businesses must address. Ethical AI deployment, data privacy, and bias mitigation are critical concerns. Organizations must ensure that AI models are trained on diverse datasets to prevent biases and maintain fairness in decision-making. Additionally, regulatory compliance and transparent AI governance frameworks are essential for building trust in AI-driven solutions.

Another challenge is workforce transformation. As AI automates routine tasks, businesses must invest in upskilling employees to work alongside AI technologies. The future workforce will require a blend of technical skills and problem-solving capabilities to effectively leverage AI-driven insights.

## 6 Future outlook: AI as a catalyst for continuous innovation

The future of AI-enabled business transformation lies in continuous innovation. As AI models become more sophisticated, businesses will increasingly adopt AI-driven decision intelligence, autonomous systems, and human-AI collaboration frameworks. The evolution of AI-powered digital twins (Park et al., 2023), generative design systems, and adaptive AI solutions will redefine industry standards and create new business opportunities.

To remain competitive, organizations must embrace AI as a strategic enabler of innovation. By integrating AI into core business functions, companies can unlock new revenue streams, enhance customer experiences, and drive operational excellence.

## 7 Conclusion

AI-enabled technologies are redefining the business landscape, offering unprecedented opportunities for automation, augmentation, and innovation. From intelligent BPM to AI-driven decision-making, businesses can harness the power of AI to achieve transformative growth. However, responsible AI deployment, ethical considerations, and workforce adaptation remain crucial for sustainable AI integration. As AI continues to evolve, its potential to revolutionize industries will only expand, solidifying its role as the cornerstone of business transformation in the digital age.
